# Evaluation of the efficacy of casein phosphopeptide-amorphous calcium phosphate on remineralization of white spot lesions in vitro and clinical research: a systematic review and meta-analysis

**DOI:** 10.1186/s12903-019-0977-0

**Published:** 2019-12-30

**Authors:** Xueling Ma, Xuandong Lin, Tengfei Zhong, Fangfang Xie

**Affiliations:** 1 Department of Stomatology, Langdong Hospital Affiliated to Guangxi Medical University, Nanning, 530021 China; 2 Department of Endodontics, Dental Hospital Affiliated to Guangxi Medical University, 10 Shuangyong Road, Nanning, 530021 China; 3 Guangxi Medical University, Nanning, 530021 China

**Keywords:** Casein phosphopeptide-amorphous calcium phosphate, Remineralization, White spot lesions, Meta-analysis

## Abstract

**Background:**

This systematic review with meta-analyses sought to answer whether casein phosphopeptide-amorphous calcium phosphate (CPP-ACP) provided a remineralizing benefit superior to that of nonintervention or placebo.

**Methods:**

Following Preferred Reporting Items for Systematic Reviews and Meta-analysis (PRISMA) guidelines, Cochrane databases, PubMed, EmBase, and Ovid up to May 20th, 2019, were scanned, only published in English. Study information extraction and methodological quality assessments were accomplished independently by two reviewers. The “Criteria for judging risk of bias in the ‘Risk of bias’ assessment tool” was used for methodological quality assessment. The continuous data was analyzed by mean difference (MD) or standardized mean difference (SMD) with a 95% confidence interval (CI). Review Manager 5.3 was used for statistical analysis. Outcome variables include quantitative light-induced fluorescence in clinical research, average surface roughness and surface microhardness in vitro.

**Results:**

There were significant differences in the quantitative light-induced fluorescence (SMD = − 0.43, 95% CI: [− 0.79, − 0.07], *P* = 0.02), average surface roughness (SMD = − 8.21, 95% CI: [− 10.37, − 6.04], *P* < 0.01), Vickers microhardness (SMD = 1.19, 95% CI: [0.72, 1.66], *P* < 0.01), and Knoop microhardness (SMD = 3.52, 95% CI: [2.68, 4.36], *P* < 0.01) between the CPP-ACP and control groups or baseline.

**Conclusion:**

Within the limitations of this meta-analysis, CPP-ACP exhibited excellent remineralization effects evaluated in clinical research and in vitro, indicating outstanding restoration of form, aesthetics, and function in treating white spot lesions.

## Background

As a major global public health issue, the incidence of dental caries is increasing in developing nations because of the ease of access to refined carbohydrates. The forming processes of caries are loops of imbalances between demineralization and remineralization, initiated by acid-producing bacteria in the micro-environment. White spot lesions (WSLs), characterized as primitive enamel surface and subsurface demineralization without cavitation [1], are developed by dental plaque accumulation, commonly owning to inadequate oral hygiene [2–4]. WSLs are commonly clinically characterized by a chalky, opaque appearance located in pits, fissures, or smooth surfaces on the teeth. As the demineralization process progresses, the intact dental surface ultimately collapses and cavitates [5]. The traditional treatment approach for carious teeth involved caries excavation and restoration, which is frequently invasive [6, 7]. However, several decades of research have culminated in “minimally invasive” approaches, emphasizing prevention rather than conventional surgical techniques. Minimally invasive dentistry utilizes programs that restore form, function, and aesthetics with minimal removal of sound tooth tissue [8]. Research indicates that demineralization can be arrested or reversed with the help of remineralization agents in WSLs or non-cavitated carious lesions [9]. Therefore, enhancing the remineralization of WSLs may be a relatively less invasive treatment of the disease [10, 11]. In conclusion, it is of great significance to explore novel agents and strategies to enhance the remineralization process.

In order to conserve tooth tissues, fluoride has been widely recommended as a remineralization agent for preventing WSLs. Despite the cariostatic effects of high concentrations of topical fluoride, its treatment capacity does have certain limitations. Because topical fluoride solutions cannot infiltrate the lesion, they do not eliminate its opaque whitish aspect [12]. Moreover, the cariostatic effects of fluoride are insufficient to manage patients with high caries risk [13] and the careless handling of fluoride may lead to adverse effects such as fluorosis [14]. To maximize the clinical significance of remineralization, a series of preventive agents containing non-fluoridated products has been developed to promote enamel remineralization.

Casein phosphopeptide-amorphous calcium phosphate (CPP-ACP), a new type of bioactive material derived from the milk protein casein, can act as a reservoir of bio-available calcium and phosphate, facilitating their precipitation on the enamel surface and thus effectively enhancing remineralization [15, 16]. Research has indicated that CPP-ACP is anticariogenic and capable of reversing the early stages of enamel lesions in vitro and in clinical research [17, 18]. However, meta-analyses comparing the remineralizing effects of CPP-ACP and placebo in clinical research and in vitro have not been performed, despite their high clinical guiding significance and laboratorial scientific research value for the exploitation of new materials. Trials only performed clinical research to evaluate the remineralization of CPP-ACP cannot be used to draw reliable conclusions, because patients may have different dietary habits or oral hygiene levels. In order to eliminate this persistent controversy and obtain more objective and accurate results, supplementary studies in vitro are necessary.

Thus, the purpose of the current study is to perform a meta-analysis including in clinical research and in vitro studies to determine whether CPP-ACP provides any remineralizing benefit superior to that of nonintervention or placebo.

## Methods

### Search strategy

An electronic systematic literature search covered the electronic databases: Cochrane Center Register of Controlled Trials, PubMed, EmBase, and Ovid in May, 2019 in English and with certain time restrictions. Additional records were identified by searching reference lists of included studies. The medical subject headings (MeSH) words and free text words were included during the search. “Casein phosphopeptide-amorphous calcium phosphate nanocomplex,” “CPP-ACP,” “GC tooth mousse,” “Recaldent,” “milk derivate,” “casein derivate,” “dental caries,” “enamel demineralization,” “white spot lesion,” “remineralisation,” “RCT,” “Randomized Controlled Trials,” “Controlled Clinical Trials,” “Equivalence Trial,” and “Pragmatic Clinical Trial” were used in combination with other strategies (more details in Additional file 1: Table S3).

Based on the titles and abstracts, initial screening of the retrieved studies was carried out. After the removal of the duplicated and obviously irrelevant studies, full texts of potential interests were reassessed and only those meeting inclusion criteria were included. This work was accomplished by two reviewers (X.M. and X.L.), independently. When any disagreement occurred, a third reviewer (F.X.) was consulted and a decision arrived at by consensus after the issues solved.

### Selection criteria

The current research followed the preferred reporting items for systematic reviews and meta-analysis (PRISMA) [19] (Additional file 1 : PRISMA Checklist, Additional file 2 : Table S3. Search strategies). The “PICO” strategy for systematic exploratory review provides guidance to the development of research contents [20]. The randomized controlled trials, retrospective and prospective studies, which were placebo-controlled or blank-controlled and had a parallel-group design were included in this research following the inclusion criteria below: (1) Participants: those for in clinical study including patients with early enamel carious had to be randomized to test or control groups. Participants for in vitro study using extracted human teeth had to utilize teeth free of any enamel defects, microcracks or caries. (2) Interventions: therapeutic dental regimes had to use remineralizing agents based on CPP-ACP. Any kind of product containing CPP-ACP could be included in this meta-analysis, such as MI Paste or Tooth Mousse. (3) Control: non-CPP-ACP therapy applied —— blank (no treatment), negative (e.g. placebo treatment and deionized water), and positive (other intervention; e.g., fluoride toothpaste). (4) Outcome: the remineralization efficacy in clinical research, average surface roughness and surface microhardness in vitro experimentation. Studies containing the follow criteria were excluded in this meta-analysis: (1) Irrelevant studies. (2) The outcomes of studies were not quantitative primary outcomes but descriptive analysis. (3) Participants of in vitro studies were non-human animal teeth, such as bovine teeth and mouse teeth.

### Quality assessment and data extraction

The Cochrane Collaboration methodology was used to assess the risk of bias of every retrieved study included. The assessment tool included random sequence generation, allocation concealment, blinding of assessment, incomplete outcome data, selective reporting, and other possible sources of bias so as to appraise the methodological quality of included studies. Bias in every study was classified as “low risk of bias,” “high risk of bias,” and “unclear risk of bias.” Cochrane Review Manager Version 5.3 (The Nordic Cochrane Centre, Copenhagen, Denmark) was used to generate risks of bias Figures.

The following information and data were extracted by two authors (X.M. and X.L.) independently from filtered studies, consisting of research features, contributor information, and major outcomes. The research features included publication date, the name of the first author, follow-up period, and type of intervention. The contributor information of in clinical experiments included demographic factors (sex and age), sample size in each group, and location of lesions, while as to the contributor information of in vitro experiments, demographic factors were replaced with tooth position. When a disagreement occurs, a third reviewer (F.X.) reaches a decision.

### Statistical analysis

The Cochrane Handbook for Systematic Reviews of Interventions was used to conduct the statistical analysis [21]. This meta-analysis of randomized control trials (RCTs) was performed to evaluate the effectiveness of CPP-ACP for WSL treatment. The data type for the outcome measurement was mainly continuous data. To avoid errors caused by different measuring instruments, the SMD was used instead of MD with a 95% CI to generalize the effectiveness of treatment in each report. *P*-values were used to test the heterogeneity across studies. For *P* < 0.05, the data is considered significantly heterogeneous. In the meantime, the degree of inconsistency of the statistical analysis was assessed by I^2^ [22]. The new quantity I^2^ has the range 0 to 100%; the values 25, 50, and 75% represent low, moderate, and high heterogeneity, respectively [23]. If all the included studies showed good homogeneity, the fixed effects model was used. When the clinical and methodological heterogeneity was high or *P* < 0.05, we used the random effects models to combine the studies [23]. RevMan statistical software version 5.3 (The Nordic Cochrane Centre, Copenhagen, Denmark) was used to conduct the statistical analyses. If there were 10 or fewer studies, publication bias was not assessed, because more than 10 studies are required to check funnel-plot asymmetry [21]. Sensitivity analysis was performed by the leave-one-out approach in this review. The analysis was carried out using STATA version 14.1 (StataCorp, College Station, Texas, USA).

## Results

### Results of the search

**Fig. 1:**
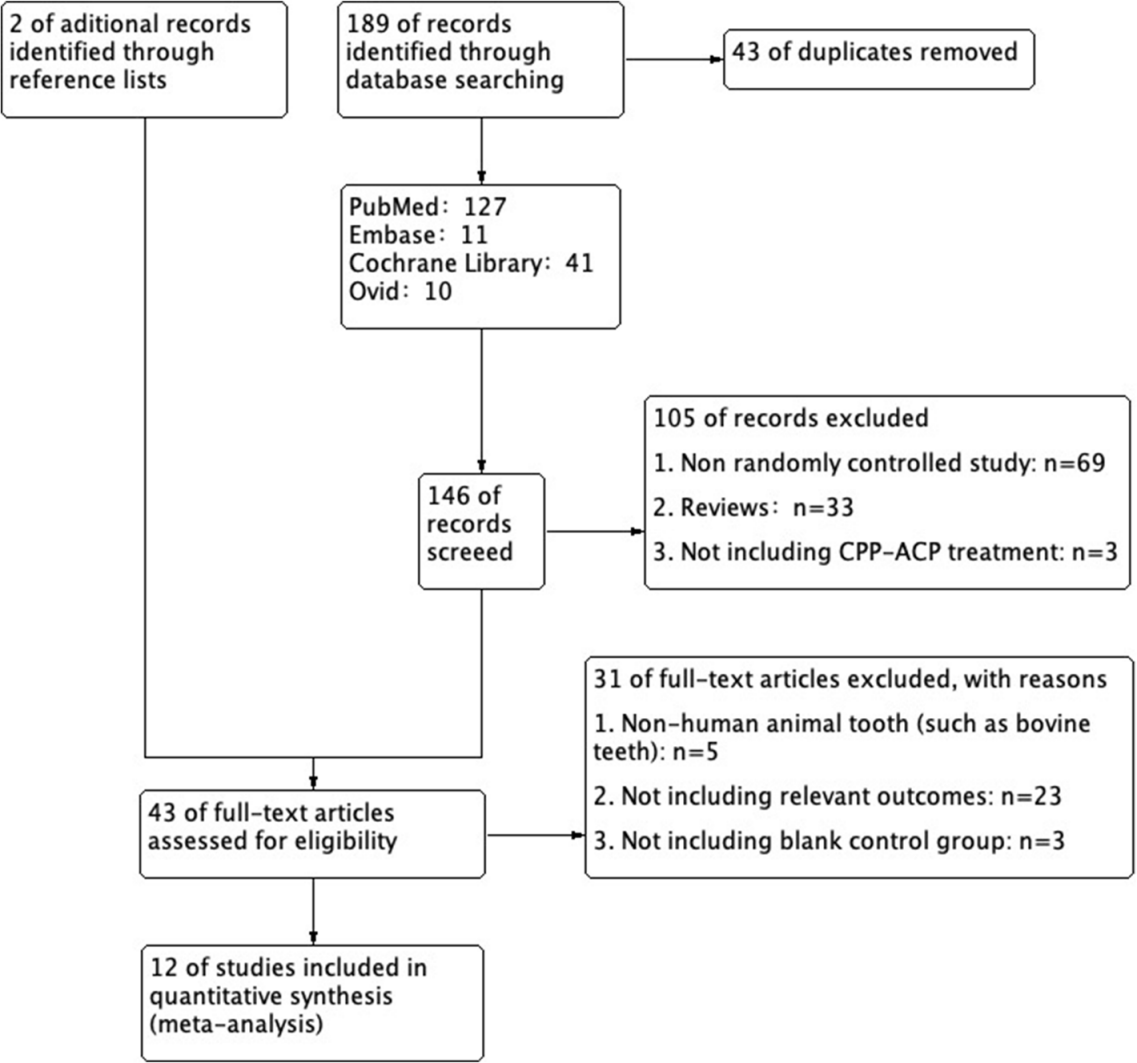
Flow diagram of the retrieved studies

Based on our retrieval search strategy, a total of 189 studies were acquired initially. From this set, 43 duplicated records were removed with the help of the reference manager EndNote X8.2. Another 105 obviously irrelevant studies were removed after scanning the titles and abstracts of these retrieved records. Two other publications were supplemented to our records through reference reading [24, 25]. Based on full-text scanning, 12 studies were eventually selected for meta-analysis among the remaining 43 studies. Many of the excluded studies met multiple exclusion criteria. A flow chart of the studies that were screened, identified, assessed for eligibility, included, and excluded in this meta-analysis is presented in Fig. 1.

### Characteristics of the included studies

More details of the characteristics of the 12 included studies are listed in Table 1 (in clinical studies) and Table 2 (in vitro studies). The publication years of these studies range from 2009 to 2017. A total of 129 patients were included in the clinical studies, with the patient ages ranging from 2.5 to 18 years. Quantitative light-induced fluorescence (QLF) measurement was performed to detect changes in fluorescence loss (ΔF). As for the in vitro study, the total number of teeth in the CPP-ACP group ranged from 6 to 15, the total number of teeth in the control group ranged from 10 to 15 and the laboratory samples came from incisors, canines, premolars, and molars. The effects of remineralization of artificial dental caries were investigated through average surface roughness and surface microhardness (SMH) which were observed by atomic force microscopy (AFM) and measured by nanoindentation respectively.

**Table 1:** Main characteristics of included trials in clinical research

First author (Year)	Country	Sample (Mean age)	Follow up	Location of lesions	Male (n, %)	CPP-ACP Group			Control Group			Outcome report
						Sample size	Type of intervention	Frequency	Sample size	Type of intervention	Frequency	
Bröchner 2014 [26]	Denmark	15.20 (n.r)^**s**^ years	4 weeks	buccal surfaces	45	22	tooth mousse	qd	28	standard fluoride toothpaste	bid	Fluorescence loss (%)
Sitthisettapong 2014 [27]	Thailand	37.51 (2.93)^**s**^ months	1 year	labial surface	54	40	fluoride toothpaste and tooth mousse	qd	39	fluoride toothpaste and placebo paste	qd	Fluorescence loss (%)

Explanations: “n.r” = “not reported,” “qd” = “once daily,” “bid” = “twice daily,” ^**s**^ Mean (standard deviation)

**Table 2:** Main characteristics of included trials in vitro

First author (Year)	Country	Follow up	Tooth position	CPP-ACP Group			Control Group			Outcome report
				Sample size	Type of intervention	Frequency	Sample size	Type of intervention	Frequency	
Zhou 2014 [15]	China	24 h	lower incisors	10	GC Tooth Mousse	tid	10	no treatment	no treatment	surface roughness (Ra, nm)
Memarpour 2015 [28]	Iran	28 d	primary canine teeth	8	GC Tooth Mousse	bid	8	no treatment	no treatment	surface roughness (Ra, nm)
Poggio 2009 [24]	Italy	36 h	upper incisors	10	GC Tooth Mousse	0, 8, 24 and 36 h	10	soft drink	0, 8, 24 and 36 h	surface roughness (Rrms, nm)
Vyavhare 2015 [29]	India	12 d	maxillary incisor teeth	6	GC Tooth Mousse	qd	6	deionized water	qd	Vickers microhardness (HV)
SoaReS 2017 [30]	India	30 d	maxillary and mandibular premolars	12	GC Tooth Mousse Plus	bid	12	no treatment	no treatment	Vickers microhardness (HV)
Rallan 2013 [31]	India	8 h	primary maxillary anterior teeth	10	GC Tooth Mousse	once	10	no treatment	no treatment	Knoop microhardness (KHN)
Carvalho 2014 [32]	Brazil	7 d	third molars	10	MI Paste Plus	qd	10	no treatment	no treatment	Knoop microhardness (KHN)
Kargul 2012 [33]	Turkey	18 h	first and second molars	15	GC Tooth Mousse	qd	15	remineralizing solutions	qd	Vickers microhardness (HV)
Memarpour 2015 [28]	Iran	28 d	primary canine teeth	10	GC Tooth Mousse	bid	10	no treatment	no treatment	Vickers microhardness (HV)
Rirattanapong 2012 [34]	Thailand	6 h	premolars	10	GC Tooth Mousse	once	10	no treatment	no treatment	Vickers microhardness (HV)
Carvalho 2013 [35]	Brazil	5 d	third molars	12	MI Paste Plus	qd	12	no treatment	no treatment	Knoop microhardness (KHN)

Explanations: “qd” = “once daily,” “bid” = “twice daily,” “tid” = “three times a day,” “h” = “hour,” “d” = “day.” GC Tooth Mousse contains 10% CPP-ACP, GC Tooth Mousse Plus contains 10% CPP-ACP and 0.2% NaF, MI Paste Plus contains 10% CPP-ACP and 900 ppm fluoride

### Assessment of methodological quality

**Fig. 2:**
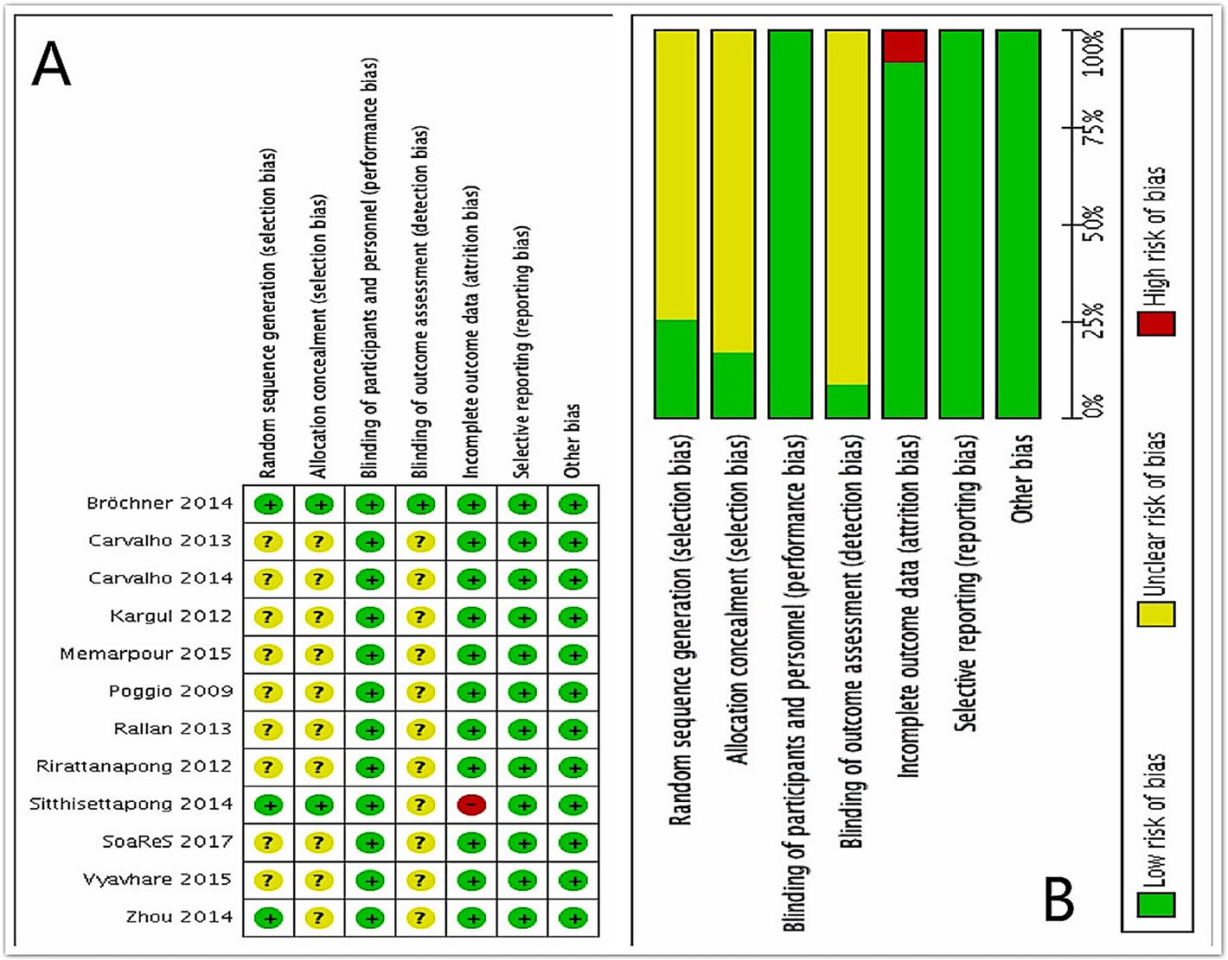
Risk of bias summary and graph. **a**) judgements about each risk of bias item for each included study. **b**) judgements about each risk of bias item presented as percentages across all included studies

Results of the assessment of methodological quality are shown in Fig. 2a and b. The judgements about each risk of bias item for each included study are presented in Fig. 2a. Figure 2b illustrates our judgements about each risk of bias item, presented as percentages across all included studies. All of the items in one study were judged as “low risk of bias [26].” All included studies had low risks of bias in selective reporting. However, one study was judged as “high risk of bias” in incomplete outcome data, on account of the loss of follow-up data [27]. Because the proportion of high risk of bias was so small, it would not seriously weaken confidence in the results. Only one study had low risk of bias in blinding of outcome assessment and the rest had unclear risks of bias. Overall, the included studies in vitro had unclear risks of random-sequence generation and allocation concealment [24, 28–35].

### Meta-analysis

#### QLF detection from in clinical experimentation

**Fig. 3:**
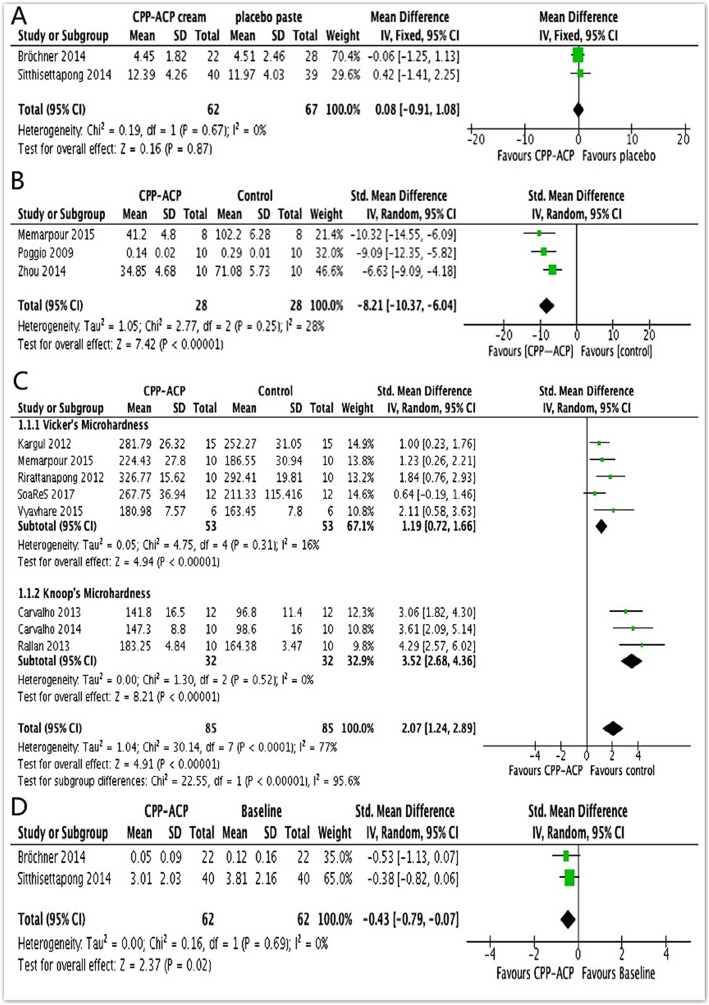
Meta-analysis in clinical and vitro experimentation. **a**) Efficacy in CPP-ACP paste and placebo paste without CPP-ACP using QLF value. **b**) Efficacy in CPP-ACP group and control group using average surface roughness value. **c**) Efficacy in CPP-ACP group and control group using SMH value. **d**) Efficacy in CPP-ACP group and baseline group without treatment using QLF value

The values of QLF were used to assess the remineralization efficacy. Both studies provided WSLs on smooth surfaces [26, 27]. After the two in clinical studies were pooled, no significant heterogeneity was found (Chi^2^ = 0.19, df = 1, *P* = 0.67, I^2^ = 0%); therefore, a fixed-effects model of analysis was used (Fig. 3a). Meta-analysis showed no significant difference between using toothpaste with CPP-ACP and using placebo paste without CPP-ACP (SMD = 0.08, 95% CI: [− 0.91, 1.08], *P* = 0.87).

#### Average surface roughness from in vitro experimentation

All three studies provided average surface roughness data and were included in the analysis [15, 24, 28]. When the data from the three studies were pooled, no significant heterogeneity was found (Chi^2^ = 2.77, df = 2, *P* = 0.25, I^2^ = 28%). Meta-analysis demonstrated a significant difference between the two groups of CPP-ACP versus the control, based on the average surface roughness measurement (SMD = − 8.21, 95% CI: [− 10.37, − 6.04], *P* < 0.00001) (Fig. 3b).

#### SMH from in vitro experimentation

There was significant heterogeneity when data from the eight studies reporting surface microhardness were pooled (Chi^2^ = 30.14, df = 7, *p* < 0.0001, I^2^ = 77%) [28–35]; therefore, a random-effects model of analysis was used (Fig. 3c). A subgroup analysis of five studies [28–30, 33, 34] including Vickers microhardness data showed that the use of CPP-ACP produced better remineralizing effects (SMD = 1.19, 95% CI: [0.72, 1.66], *P* < 0.00001). As to the remaining three studies [31, 32, 35] including Knoop microhardness data, the subgroup analysis also showed a significant difference between the CPP-ACP and control groups (SMD = 3.52, 95% CI: [2.68, 4.36], *P* < 0.00001).

#### Sensitivity analysis and publication bias

**Fig. 4:**
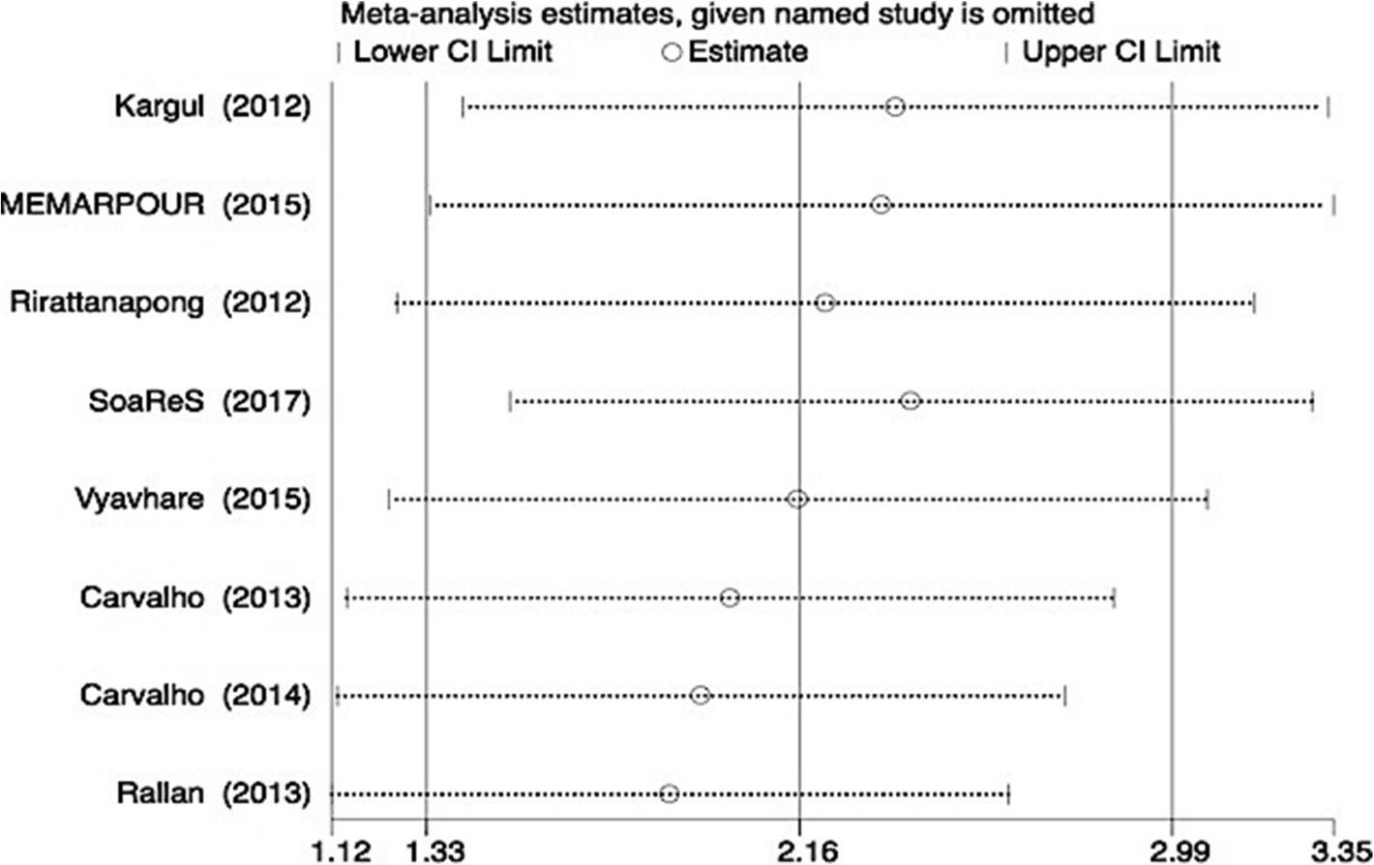
Outcome of sensitivity analysis of SMH in vitro

The leave-one-out approach was used to assess the sensitivity of meta-analysis. When individual studies are eliminated in turn, all results are consistent with the meta-analysis results using all studies and the directions of the polled estimates of SMH do not vary considerably (Fig. 4). This means that the meta-analysis had good reliability and stability. Publication bias was not evaluated for these results because the detection of funnel plot asymmetry requires more than 10 studies.

## Discussion

The use of the proposed minimally invasive technique can not only induce recovery of the natural tooth appearance but also promote enamel remineralization in depth, so it may be considered a potential alternative to conventional operative treatment. The proposed minimally invasive technique mainly utilizes a combined approach of microabrasion and enamel remineralization [36]. Fluoride therapy has long been considered as the base non-invasive treatment for early carious lesions, although many defects exist in the use of fluoride. The low permeability of fluoride hinders elimination of the opaque whitish aspect, thus compromising esthetics [12]. Biotoxicity from the inappropriate use of fluoride may have adverse effects such as fluorosis [14]. CPP-ACP, a nanocomplex derived from milk, can restrict calcium phosphate growth to the critical size required for nucleation and subsequent precipitation [37]. In addition to its high safety level, CPP-ACP has demonstrated anticariogenic potential in the laboratory and human in situ experiments [38–40]. In conclusion, CPP-ACP has the advantages of maximum tooth substance conservation and excellent acceptance by patients. Thus, the aim of this study is to comprehensively evaluate the biological remediation effects of CPP-ACP on patients with WSLs in clinical and on artificial demineralized models in vitro. Several measurement indicators, including QLF detection, average surface roughness, and surface microhardness, can provide a comprehensive assessment of CPP-ACP in terms of form, aesthetics, and function restoration, respectively, which are important requirements for minimally invasive dentistry [8].

Experimental results in clinical [26, 27] indicate that, compared with placebo paste without CPP-ACP, CPP-ACP paste showed no significant advantage for the prevention of enamel demineralization. This can be attributed to the intervention of fluoride in the control group, which can facilitate calcium and phosphate diffusion into the WSLs to partially remineralize the crystalline structures. In order to remove this interfering factor, supplementary analysis without fluoride is necessary. Then the supplementary comparison between the CPP-ACP and baseline groups was performed. As shown in Fig. 3d, after the data from the two studies were pooled, no significant heterogeneity was found (Chi^2^ = 0.16, df = 1, *P* = 0.69, I^2^ = 0%). Meta-analysis demonstrated a significant difference between the CPP-ACP and baseline groups, as assessed by QLF values (SMD = − 0.43, 95% CI: [− 0.79, − 0.07], *P* = 0.02). QLF meets the basic requirement for detection, quantification, and monitoring of carious lesions and is widely used in clinical trials to monitor WSLs as well as to investigate the efficacy of bioremediation. Based on the principal of detecting changes in fluorescence correlating to mineral loss, caused by caries destruction of enamel, QLF measurement can reflect changes in tooth enamel form [41]. Our results showed a significant improvement in WSLs regression by using CPP-ACP as assessed with QLF. In conclusion, this meta-analysis showed a noticeable improvement of WSLs remineralization as assessed by QLF, which means that CPP-ACP can accomplish enamel form recovery.

The analysis of average surface roughness [15, 24, 28] indicated that, with the help of CPP-ACP, the roughness of the enamel surface was decreased to a statistically significant degree. Atomic force microscopy (AFM) permits observation of the nanoscale appearance of softened enamel surfaces. The average surface roughness not only refers to aesthetic properties, but also reflects bacterial adhesion and plaque formation potential in the oral environment [42]. The enamel surface roughness measurement results confirmed that the center areas of enamel prisms were restored gradually by CPP-ACP induction, still according to the orientations of mineralized fibrils, until the enamel surface became flat and smooth [15]. This analysis of average surface roughness performed by AFM suggests that CPP-ACP has excellent ability to repair and smooth the surface of enamel, ultimately acquiring desirable aesthetic effects.

Regarding the assessment of function restoration, the analysis of SMH in vitro was performed. The SMH test offers a relatively simple, rapid, and non-destructive approach in demineralization and remineralization studies [43]. Different description units of SMH (Vickers hardness and Knoop hardness) could all induce heterogeneity within one study when comparing outcomes; therefore, we conducted subgroup analysis according to different testing methods, such that the heterogeneity reduced I^2^ from 77 to 16% and 0%, respectively (Fig. 3c). This indicated that the different testing methods were the main factors inducing heterogeneity, so the subgroup analysis permitted comparison of the outcomes. After CPP-ACP treatment and remineralization, the mean SMH values increased significantly compared to those of the control group, whether measured with a Vickers microhardness tester or Knoop hardness tester. This result can be attributed to the mineralization induction of CPP-ACP: after the localization of ACP at the enamel surface, free calcium and phosphate ions were buffered, thereby helping to maintain a state of supersaturation with respect to tooth minerals, depressing enamel demineralization, and promoting remineralization [37, 44].

Some potential limitations of the study should be addressed. Our meta-analysis, including average surface roughness and surface microhardness, was based on in vitro environments, which was not reproduced in clinical; therefore, many limitations were unavoidable. For instance, the effects of salivary enzymes, proteins, pellicle, dental plaque, and additional fluoride sources on demineralization and remineralization cycles in the oral environment were not included [45]. However, from another perspective, considering the good control of interfering factors for in vitro studies, the results may be more stable and convincing. Furthermore, there is considerable risk of lowering the quality of the evidence in surface microhardness analysis. The CPP-ACP group included both “GC Tooth Mousse Plus” and “MI Paste Plus,” which contains a small amount of fluoride, as expounded in Table 2 [37, 44]. However, certain studies [46, 47] have demonstrated that CPP-ACP combined with fluorides achieved no clinical advantage, so we combined the three experiments into our meta-analysis. Despite these limitations, the ideal treatment effect of CPP-ACP for WSLs remained evident.

Some guidelines may be helpful in clinical operation and future studies. When treating patients with WSLs, dentists are recommended to prioritize CPP-ACP, especially in children whose risk factors can be controlled adequately. Considering its preferable aesthetics effect, CPP-ACP is highly recommended to those who, with high aesthetic requirements, developed WSLs after orthodontic treatment. But one thing still needs to be pointed out, CPP-ACP is not of zero risk and absolute security. There have been reports of patient deaths from allergic reaction. When applied in clinic, we need to pay special attention to their system history and allergies. To evaluate the effect of remineralization in a more comprehensive way, further studies of CPP-ACP, especially in combination with new detection indexes both in clinical and in vitro, remain necessary. Considering the good advantage of CPP-ACP over traditional fluoride, the subsequent comparison between CPP-ACP and fluoride must be inevitable.

## Conclusions

Based on this study’s results analyzing in vitro and in clinical data, CPP-ACP exhibited excellent remineralization of WSLs compared to the other groups or baseline, with greater percentages of WSL regression, lower enamel surface roughness, and the highest surface microhardness recovery. This indicates that CPP-ACP can effectively restore form, aesthetics, and function. Therefore, CPP-ACP seems effective for the remediation of WSLs.

## Supplementary information

**Additional file 1:** **Table S3.** Search stratdgies.

**Additional file 2:** PRISMA Checklist

## Data Availability

The datasets used and/or analyzed during the current study are available from the corresponding author on reasonable request.

## Abbreviations

AFM: Atomic force microscopy
CI: Confidence interval
CPP-ACP: Casein phosphopeptide-amorphous calcium phosphate
MD: Mean difference
QLF: Quantitative light-induced fluorescence
RCTs: Randomized control trials
SMD: Standardized mean difference
SMH: Surface microhardness
WSLs: White spot lesions
ΔF: Fluorescence loss
