# Supplementary material for: Evaluation of the efficacy of casein phosphopeptide-amorphous calcium phosphate on remineralization of white spot lesions in vitro and clinical research: a systematic review and meta-analysis
Source: BMC Oral Health. 2019 Dec 30;19:295. doi: 10.1186/s12903-019-0977-0 (PMC6937959; doi:10.1186/s12903-019-0977-0)
Supplement: Supplementary file 2 — Additional file 2: PRISMA Checklist [file 12903_2019_977_MOESM2_ESM.doc]

**Table 3 Search strategies.**

| **Database** | **Period of Search** | **Search strategies** |
| --- | --- | --- |
| **PubMed** | January 1st, 1990 until  May 20th, 2019 | #1 (((((("Fluoride "[Mesh]) OR "Sodium Fluorides") OR "Sodium Fluorides") OR "Fluoristat") OR "Fluorides, Sodium") OR "Fluoristats") OR "Fluoride, Sodium"  #2 (((((("casein phosphopeptide-amorphous calcium phosphate nanocomplex" [MeSH]) OR “casein phosphopeptide-amorphous calcium phosphate nanocomplex”) OR “CPP-ACP”) OR “tooth mousse”) OR “GC tooth mousse”) OR “Recaldent”  #3 (((((("Dental Caries"[Mesh]) OR “Dental Decay”) OR “Caries”) “enamel demineralization”) OR “white spot lesion”) OR “remineralisation”) OR “tooth decay”  #4 #1 AND #2  #5 #3 AND #4 |
| **Embase** | January 1st, 1990 until  May 20th, 2019 | #1 exp casein/  #2 exp fluoride/  #3 #1 AND #2 |
| **Cochrane Library** | January 1st, 1990 until  May 20th, 2019 | 1. exp Caseins/ 2. exp CPP-ACP/ 3. exp milk derivate / 4. exp Caseins/ 5. exp Fluorides/ 6. exp Fluorides, Topical/ 7. exp Sodium Fluorides/ 8. 5 or 6 or 7 9. 1 or 2 or 3 or 4 10. 8 and 9 |
| **Ovid** | January 1st, 1990 until  May 20th, 2019 | 1. exp Caseins/ 2. exp Fluoride/ 3. exp Caries/ 4. 1 and 2 and 3 |
